# The Radical Scavenger NZ-419 Suppresses Intestinal Polyp Development in *Apc*-Mutant Mice

**DOI:** 10.3390/jcm9010270

**Published:** 2020-01-18

**Authors:** Yurie Kurokawa, Gen Fujii, Susumu Tomono, Shingo Miyamoto, Takahiro Hamoya, Maiko Takahashi, Takumi Narita, Masami Komiya, Masaki Kobayashi, Yoshikazu Higami, Michihiro Mutoh

**Affiliations:** 1 Epidemiology and Prevention Division, Research Center for Cancer Prevention and Screening, National Cancer Center, Tokyo 104-0045, Japan; have.a.foresight.9696@gmail.com (Y.K.); s-miyamoto@po.kyoundo.jp (S.M.); thamoya@ncc.go.jp (T.H.); mymymai999@gmail.com (M.T.); tanarita@ncc.go.jp (T.N.); mkomiya@ncc.go.jp (M.K.); 2 Laboratory of Molecular Pathology and Metabolic Disease, Faculty of Pharmaceutical Sciences, Tokyo University of Science, Chiba 278-8510, Japan; kobayashim@rs.tus.ac.jp (M.K.); higami@rs.noda.tus.ac.jp (Y.H.); 3 Central Radioisotope Division, National Cancer Center Research, Tokyo 104-0045, Japan; gfujii@ncc.go.jp; 4 Department of Microbiology and Immunology, Aichi Medical University, Nagakute, Aichi 480-1195, Japan; tomono@aichi-med-u.ac.jp; 5 Department of Cancer Cell Research, Sasaki Institute, Sasaki Foundation, Tokyo 101-0062, Japan; 6 Translational Research Center, Research Institute of Science and Technology, Tokyo University of Science, Chiba 278-8510, Japan; 7 Division of Carcinogenesis and Cancer Prevention, National Cancer Center Research Institute, Tokyo 104-0045, Japan

**Keywords:** *Apc*-mutant mice, NZ-419, Nrf2, hydroxyl radical, cancer chemoprevention

## Abstract

Colorectal cancer is the fourth leading cause of cancer death worldwide, and it is important to establish effective methods for preventing colorectal cancer. One effective prevention strategy could be the use of antioxidants. However, the role of the direct antioxidative function of antioxidants against carcinogenesis has not been clarified. Thus, we aimed to determine whether the direct removal of reactive oxygen species by a hydroxyl radical scavenger, NZ-419, could inhibit colorectal carcinogenesis. NZ-419 is a creatinine metabolite that has been shown to be safe and to inhibit the progression of chronic kidney disease in rats, and it is now under clinical development. In the present study, we demonstrated that NZ-419 eliminated reactive oxygen species production in HCT116 cells after H_2_O_2_ stimulation and suppressed H_2_O_2_-induced Nrf2 promoter transcriptional activity. The administration of 500 ppm NZ-419 to *Apc*-mutant Min mice for 8 weeks resulted in a decrease in the number of polyps in the middle segment of the small intestine to 62.4% of the value in the untreated control (*p* < 0.05 vs. control group). As expected, NZ-419 treatment affected the levels of reactive carbonyl species, which are oxidative stress markers in the serum of Min mice. Suppression of the mRNA levels of the proliferation-associated factor *c-Myc* was observed in intestinal polyps of Min mice after NZ-419 treatment, with a weak suppression of epithelial cell proliferation assessed by proliferation cell nuclear antigen (PCNA) staining in the intestinal polyps. This study demonstrated that NZ-419 suppress the development of intestinal polyps in Min mice, suggesting the utility of radical scavenger/antioxidants as a cancer chemopreventive agent.

## 1. Introduction

Colorectal cancer (CRC) currently accounts for approximately 8% of all cancer deaths and is the fourth leading cause of cancer deaths worldwide [1]. Thus, it is important to establish effective methods for preventing CRC. One potentially effective prevention strategy could be the use of chemopreventive agents such as antioxidants.

However, there are several problems to be solved. To date, many epidemiological, clinical and experimental studies have suggested that the inhibition of oxidative stress may reduce the risk of CRC. However, some antioxidants may exert their antineoplastic activity not only through antioxidative function but also through multifunctional actions. For instance, ω3 polyunsaturated fatty acids (PUFAs) such as docosahexaenoic acid (DHA, C22:6) and eicosapentaenoic acid (EPA, C20:4), which are major components of fish oil, have been evaluated for their preventive effects against colon carcinogenesis using rodent models [2]. A recent phase III randomized, double-blind, placebo-controlled trial of EPA (2 g daily for 6 months) for familial adenomatous polyposis (FAP) patients demonstrated a 22.4% reduction in polyp number (*p* = 0.012) and a 29.8% decrease in the sum of polyp diameters (*p* = 0.027) in the EPA-treated group [3]. PUFAs are highly peroxidizable and may reduce reactive oxygen species (ROS) levels. However, it also inhibits cyclooxygenase (COX) activity and acts as a direct ligand for G protein-coupled receptors (GPCRs) [2,3]. As another example, lutein has been reported to have a superior antioxidant ability to scavenge free radicals compared with other carotenoids. Lutein is also an anticarcinogenic reagent that has the potential to modulate cell growth [4,5] and apoptosis signaling [6]. There are more antioxidant phytochemicals that show both antioxidant function and cancer preventive function [7,8,9,10]. From these reports, antioxidants have potential as cancer chemopreventive agents in the colorectum, but the proportional contribution of antioxidative function to carcinogenesis has not yet been clarified.

To obtain direct evidence that the use of antioxidants could be an effective prevention strategy, we should show whether the direct deletion of ROS could inhibit colorectal carcinogenesis or not. Previously, mesalamine (5-aminosalicylic acid, 5-ASA) was shown to directly scavenge peroxynitrite, and treatment with 5-ASA at 0.1 and 1.0 mM was found to significantly inhibit DNA strand breaks induced by the peroxynitrite generator 3-morpholinosydnonimine [11]. Moreover, studies have demonstrated that 5-ASA is associated with an overall decrease in the risk of developing CRC in ulcerative colitis patients. In the case of animal experiments, 5-ASA administrations to Min mice (FAP model mice that have an *Apc* mutation and develop intestinal polyps) at doses of 500, 2400, 4800, and 9600 parts/million (ppm) failed to show direct chemosuppressive activity against the development of intestinal polyps [12].

**Figure 1:**
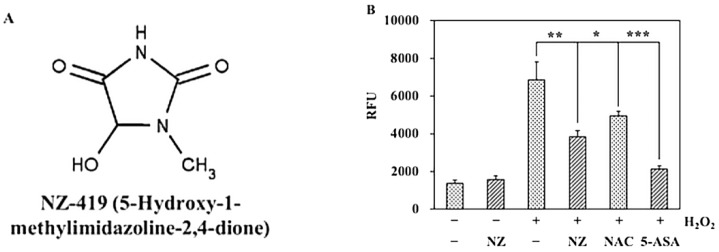
NZ-419 eliminates reactive oxygen species (ROS) production in HCT116 cells. (**A**) Chemical structure of NZ-419. (**B**) HCT116 cells were treated with 1 mM NZ-419 (NZ), 5 mM *N*-Acetyl-l-cysteine (NAC), 100 µM 5-aminosalicylic acid (5-ASA) for 30 min and then treated with 0.5 mM H_2_O_2_ and further incubated for 10 min to evaluate the amount of ROS production. NAC and 5-ASA are positive controls. Data are presented as the means ± SD (*n* = 3). Asterisks indicate significant difference from the untreated control group at * *p* < 0.05, ** *p* < 0.01, and *** *p* < 0.005. The data are representative data obtained from more than three independent experiments.

We searched for a drug that could be administered orally and possessed ROS scavenging activity and found that 5-hydroxy-1-methylhydantoin (NZ-419; Figure 1A), a creatinine metabolite, has hydroxyl radical (·OH) scavenging ability and has been shown to inhibit the progression of chronic kidney disease in rats [13,14]. Creatinine is a well-known major intrinsic ·OH scavenger, and its metabolites also exist in the body with low toxicity. NZ-419 can potentially be a therapeutic agent against progressive chronic renal failure at chronic kidney disease (clinical Stages 3 and 4) and is now under clinical development (in Phase II clinical trial).

In the present study, we confirmed the ·OH scavenging activity of NZ-419 in human CRC cells. Furthermore, we examined the suppressive effects of NZ-419 on intestinal polyp formation administration in Min mice.

## 2. Materials and Methods

### 2.1. Chemicals

NZ-419 was kindly provided by Nippon Zoki Pharmaceutical Co., Ltd. H_2_O_2_ was purchased from Wako Pure Chemical Industries (Osaka, Japan). *N*-Acetyl-l-cysteine was purchased from Sigma-Aldrich (St. Louis, MO, USA). 5-Aminosalicylic acid was purchased from Tokyo Chemical Industry (Tokyo, Japan). Acrolein, crotonaldehyde, dansyl hydrazine (DH), glyoxal, 2,4-decadienal (DDE), heptadecanal, hexadecanal, 2,4-nonadienal (NDE), octadecanal, 2-octenal, pentadecanal, tetradecanal and 2-undecenal were also purchased from Tokyo Chemical Industry. Acetaldehyde, *p*-Toluenesulfonic acid (*p*-TsOH) and the RCs, including propanal, pentanal, butanal, 2-hexenal, hexanal, 2-heptenal, heptanal, octanal, 2-nonenal, nonanal, decanal, undecanal, dodecanal and tridecanal, were obtained from Sigma-Aldrich. 4-Hydroxy-2-hexenal (HHE), 4-hydroxy-2-nonenal (HNE) and 4-oxo-2-nonenal (ONE) were purchased from Cayman Chemical Company (Ann Arbor, MI, USA). *p*-Benzyloxybenzaldehyde (*p*-BOBA) was purchased from Wako Pure Chemical Industries. 8-Heptadecenal (8-HpDE), 8,11-heptadecadienal (8,11-HpDDE) and 8,11,14-heptadecatrienal (8,11,14-HpDTE) were synthesized using a previously described method [15,16]. Secosterol-A and B were synthesized according to a procedure reported by Wentworth et al. [17]. Stock solutions of the reactive carbonyl species (RCs) and an internal standard (IS) (*p*-BOBA, 10 µM) were prepared separately in acetonitrile and stored at −20 °C prior to use.

### 2.2. Cell Culture

HCT116 human colon adenocarcinoma cells were purchased from the American Type Culture Collection (Manassas, VA, USA). HCT116 was maintained in DMEM supplemented with 10% heat-inactivated fetal bovine serum (FBS; HyClone Laboratories Inc., Logan, UT, USA) and antibiotics (100 µg/mL streptomycin and 100 U/mL penicillin) at 37 °C with 5% CO_2_.

### 2.3. Animals

Male C57BL/6-Apc^Min/+^ mice (Min mice) were purchased from Jackson Laboratory (Bar Harbor, ME, USA). The mice (*n* = 3–4) were housed in plastic cages with sterilized softwood chips as bedding in a barrier-sustained animal room maintained at 24 ± 2 °C and 55% humidity under a 12 h light/dark cycle. NZ-419 was mixed with the basal diet AIN-76A (Japan CLEA, Tokyo, Japan) at a dose of 500 or 1000 ppm every 2 weeks.

### 2.4. Animal Experimental Protocol

Nine male Min mice aged 5 weeks were given 0, 500 or 1000 ppm NZ-419 for 8 weeks. All animals housed in the same cage were included in the same treatment group. Food and water were available ad libitum. We used a hanging bait box that will not spills diets from it and will not allow the mice to urinate inside, Moreover, it is easy to measure the weight of food, which allowed us to easily calculate an accurate amount of food intake/cage. The animals were observed daily for clinical symptoms and mortality. Body weight and food consumption were measured weekly.

At the sacrifice time point, the mice were anesthetized, and blood samples were collected from their abdominal veins. Their intestinal tracts were removed and separated into small intestine, cecum and colon. The first 4 cm of the small intestine was separated and considered the proximal segment; and the rest of the small intestine was divided in half to produce the middle and distal segments. The polyps in the proximal segments were counted and collected under a stereoscopic microscope at the time of sacrifice. The remaining intestinal mucosa (non-polyp part) was removed by scraping, and the specimens were stored at −80 °C until quantitative real-time PCR analysis. The other regions were opened longitudinally and fixed flat between sheets of filter paper in 10% buffered formalin. Later, polyp number, size and intestinal distribution were assessed with a stereoscopic microscope. All experiments were performed according to the “Guidelines for Animal Experiments in the National Cancer Center” and were approved by the Institutional Ethics Review Committee for Animal Experimentation of the National Cancer Center. The animal protocol was designed to minimize pain and discomfort to the animals. The animals were acclimatized to laboratory conditions for more than one week prior to experimentation. All animals were euthanized by isoflurane overdose for tissue collection.

### 2.5. Measurement of Intracellular ROS

HCT116 cells (2 × 10^4^ cells/well) were cultured in 96-well black plates (Sigma-Aldrich) for 24 h. Cells were treated with 1 mM DCFH-DA (Cell Biolabs, Inc., San Diego, CA, USA) and incubated for 60 min at 37 °C in the dark. Cells were then rinsed three times with PBS and treated with 1 mM NZ-419 (NZ), 5 mM *N*-Acetyl-l-cysteine (NAC), 100 μM 5-aminosalicylic acid (5-ASA) for 30 min followed by incubation with 0.5 mM H_2_O_2_ for 10 min at 37 °C in the dark. Fluorescence intensity was determined by iMark^TM^ Microplate Absorbance Reader (Bio-Rad, Hercules, CA, USA). The excitation and emission wavelengths were 485 nm and 535 nm, respectively. Data are expressed as the means ± standard deviation (SD) (*n* = 3).

### 2.6. Luciferase Assays for Nrf2 Promoter Transcriptional Activity

HCT116 human colon cancer cells were transfected with pNrf2/ARE-Luc (Promega, Madison, WI, USA) reporter plasmids using Polyethylenimine MAX MW 40,000 (PolyScience, Warrington, PA, USA). Then, transfectants stably expressing Nrf2-Luc, to measure Nrf2 transcriptional activity, were obtained after treatment with hygromycin and cloning. The cells were referred to as HCT116-Nrf2-Luc cells. HCT116-Nrf2-Luc cells were seeded in 96-well plates (2 × 10^4^ cells/well). After 24 h incubation, the cells were treated with 1, 10, 100 or 10,000 μM NZ-419 with or without 40 μM H_2_O_2_ for 24 h. Firefly luciferase activity levels were determined using the Bright GLO Luciferase Assay System (Promega). The basal Nrf2 luciferase activity in the control was set as 1.0. Data are expressed as the means ± SD (*n* = 3).

### 2.7. Extraction and Analysis of Reactive Carbonyl Species

Mouse serum samples were homogenized in sodium phosphate buffer (50 mM, pH 7.4) containing 0.5 mM EDTA and 20 μM BHT. The serum homogenates were mixed with an IS (*p*-BOBA) (20 pmol) and 400 μL of a chloroform/methanol (2:1, *v*/*v*) solution. The resulting mixture was vigorously agitated for 1 min and then centrifuged at 15,000 rpm for 10 min, and the organic phase was collected. The remaining precipitate and aqueous phases were then mixed with 400 μL of the chloroform/methanol solution (2:1, *v*/*v*), and the resulting mixture was centrifuged at 15,000 rpm for 10 min to obtain the organic phase. The combined organic phases were mixed with 100 μL of acetonitrile containing 50 μg of DH and 10 μg of *p*-toluenesulfonic acid and incubated for 4 h at ambient temperature in the absence of light. The mixtures were then evaporated to dryness in vacuo to yield the corresponding derivatized residues. These residues were dissolved in 200 μL of acetonitrile, and 5 μL of each sample was injected into the LC/MS system. The LC/MS conditions were modified from the previous analysis [18,19]. The RC-DH derivatives were separated on a CORTECS UPLC C18 column (1.6 μm, 100 × 2.1 mm, Waters) connected to a Shimadzu UHPLC Nexera X2 system (Shimadzu) and a Sciex TripleTOF 5600+ system with Duospray^TM^ ion source (Sciex). Mobile phase A consisted of a 0.1% (*v*/*v*) solution of formic acid in water, and mobile phase B consisted of a 0.1% (*v*/*v*) solution of formic acid in acetonitrile. The linear gradient conditions were as follows: 20% B at 0 min, 100% B at 5 min, 100% B at 12 min and 20% B at 12.01 min, followed by a 2.99 min equilibration time. The system was operated at a constant flow rate of 0.4 mL/min for all of the analyses. The instrument parameters for positive-ion mode were as follows: curtain gas, 35 psi; collision arbitrary units; ionspray voltage, 5500 V; temperature, 350 °C; ion source gas 1, 60 psi; ion source gas 2, 60 psi; declustering potential, 80 V; collision energy, 45 V; collision energy spread, 15 V. The RC-DH derivatives were detected using the MRMHR mode. The details regarding RCs profiling strategy have been described previously [18,19].

### 2.8. Quantitative Real-Time Polymerase Chain Reaction (qPCR) Analyses

Total RNA was isolated using TRIzol (Invitrogen, Grand Island, NY, USA), and 1 µg aliquots in a final volume of 20 µL were used for cDNA synthesis using a High Capacity cDNA Reverse Transcription Kit (Applied Biosystems, Foster City, CA, USA). Real-time PCR was carried out using the CFX96/384 PCR Detection System (Bio-Rad) and Fast Start Universal SYBR Green Mix (Roche Diagnostics, Mannheim, Germany) according to the manufacturers’ instructions. The primer sequences were as follows: 5′-GCCCGCGCCCAGACAGGATA, 3′-GCGGCGGCGGAGAGGA (c-Myc), 5′-CTGGTGTTTGAGCATGTAGACC, 3′-GATCCTTGATCGTTTCGGCTG (Cdk4), 5′-TTGTCTCCTGCGACTTCA, 3′-CACCACCCTGTTGCTGTA (GAPDH). To assess the specificity of each primer set, the melting curves of the amplicons generated by the PCR reactions were analyzed.

### 2.9. Immunohistochemical Staining

The middle segments of the small intestines were fixed, embedded, and sectioned using the Swiss roll technique for further immunohistochemical examination with the avidin–biotin immunoperoxidase procedure. Monoclonal mouse anti-PCNA antibody (Santa Cruz Biotechnology, Santa Cruz, CA, USA) was used at a 1:200 dilution. As the secondary antibody, biotinylated anti-mouse IgG, absorbed with horse serum (Vector Laboratories, Burlingame, CA, USA) was employed at a 1:200 dilution. Staining was performed using avidin–biotin reagents (Vectastain ABC reagents; Vector Laboratories, Burlingame, CA, USA), 3,3′-diaminobenzidine and hydrogen peroxide, and the sections were counterstained with hematoxylin to facilitate orientation. All polyps were imaged at 20× magnification, and the PCNA-positive cell ratio (%) was calculated as follows: number of PCNA-stained nuclei in the polyp/number of nuclei in the polyp × 100.

### 2.10. Statistical Analyses

All results are expressed as the means ± SD, and all statistical analyses were performed using Student’s *t* tests, except for the analyses of the number of intestinal polyps and the luciferase assay data. The numbers of intestinal polyps and the luciferase assay data were analyzed using Dunnett’s test. Differences were considered statistically significant at * *p* < 0.05, ** *p* < 0.01 and *** *p* < 0.005.

## 3. Results

### 3.1. NZ-419 Eliminated ROS in HCT116 Cells

To confirm the ability of NZ-419 to eliminate ROS in HCT116 cells, the oxidation-sensitive fluorescent probe H_2_DCFDA was used to monitor the production of both hydrogen peroxide and cytosolic superoxide anions. After treatment with H_2_O_2_ for 10 min, 1 mM NZ-419 significantly decreased the DCF fluorescence intensity to 55.9% of the untreated control value, respectively. In addition, NAC and 5-ASA, well-known ROS scavengers used as a positive control, decreased to 72.1%, and 31.0% (Figure 1B).

### 3.2. NZ-419 Suppressed H_2_O_2_-Induced Nrf2 Promoter Transcriptional Activity

**Figure 2:**
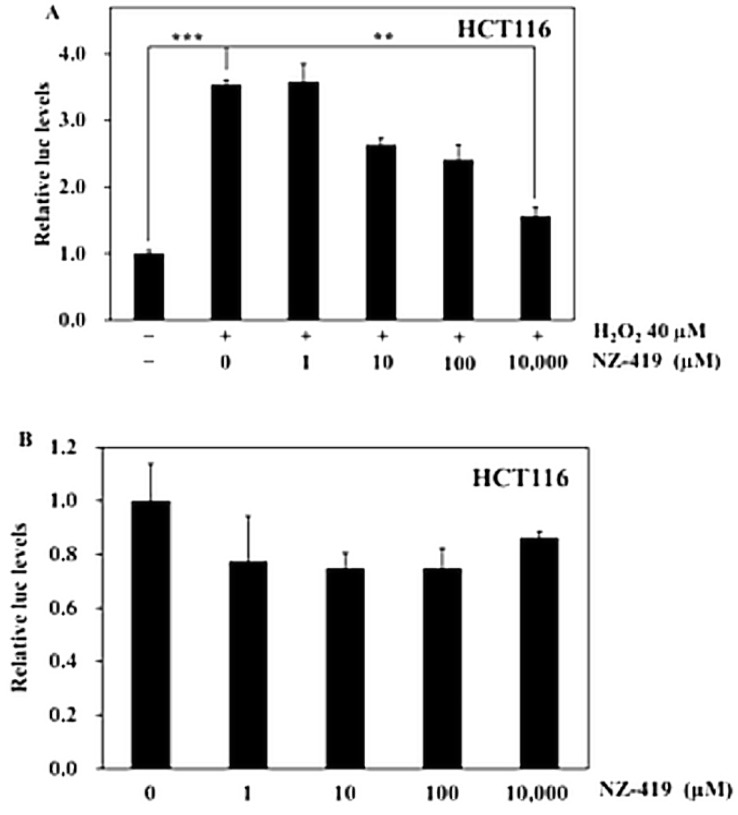
NZ-419 suppresses H_2_O_2_-induced Nrf2 promoter transcriptional activity. HCT116 cells were treated with NZ-419 (10,000 µM) and incubated for 30 min at 37 °C. After incubation, cells were treated with 40 μM H_2_O_2_ for 24 h (**A**), or for 24 h without H_2_O_2_. (**B**) The basal luciferase activity level of the control was set as 1.0. The data are presented as the means ± SD (*n* = 3). ** *p* < 0.01, *** *p* < 0.005 vs. 0 control. The data are representative data obtained from more than three independent experiments.

To confirm the antioxidative function of NZ-419, the effects of NZ-419 on nuclear factor erythroid-2 related factor 2 (Nrf2) promoter transcriptional activity in response to oxidative stress were measured. We used H_2_O_2_ as oxidant, which clearly increased promoter transcriptional activity by 354% (*p* < 0.005) compared with the control. Moreover, treatment of HCT116 human colon cancer cells with 10,000 µM NZ-419 for 24 h decreased H_2_O_2_-induced Nrf2 promoter transcriptional activity by 56% (*p* < 0.01), respectively, compared with the control value in cells treated with H_2_O_2_ alone (Figure 2A). The cells only treated with NZ-419 didn’t decrease basal Nrf2 promoter transcriptional activity (Figure 2B).

### 3.3. Suppression of Intestinal Polyp Formation in Min Mice by NZ-419

**Figure 3:**
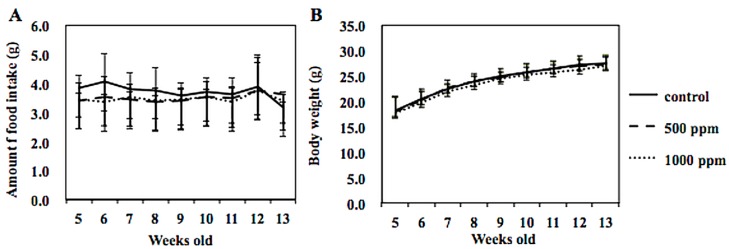
Amount of food intake and body weight changed throughout the experimental period. Amount of food intake /mouse/day (**A**) and body weight of each mouse (**B**) were measured weekly.

**Figure 4:**
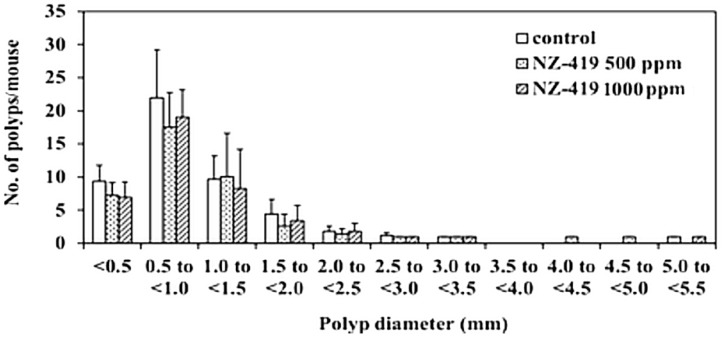
NZ-419 inhibited the size of intestinal polyps. The size distributions of the intestinal polyps in the basal control diet and NZ-419-treated groups are shown.

Administration of 500 or 1000 ppm NZ-419 to Min mice for 8 weeks did not affect food intake (Figure 3A), body weight (Figure 3B) or clinical symptoms throughout the experimental period. There were no differences in average daily food intake among the 0, 500 and 1000 ppm groups of Min mice. In addition, no changes in organ weights attributable to toxicity were observed. Table 1 summarizes the data regarding the number and distribution of intestinal polyps in the untreated control group and NZ-419-treated groups. The majority of polyps developed in the small intestine, while only a few developed in the colon. Treatment with 500 ppm NZ-419 decreased the number of polyps in the middle segment of the small intestine to 62.4% of the value in the untreated controls (*p* < 0.05 vs. control group). No significant differences in the numbers of polyps were observed in the other segment of the small intestine or the colon. Figure 4 shows the size distributions of the intestinal polyps in the untreated control group and the NZ-419-treated groups. The majority of polyps were approximately 0.5–1.0 mm in diameter.

### 3.4. The Levels of Oxidative Stress-Related Markers in Min Mice by NZ-419

**Figure 5:**
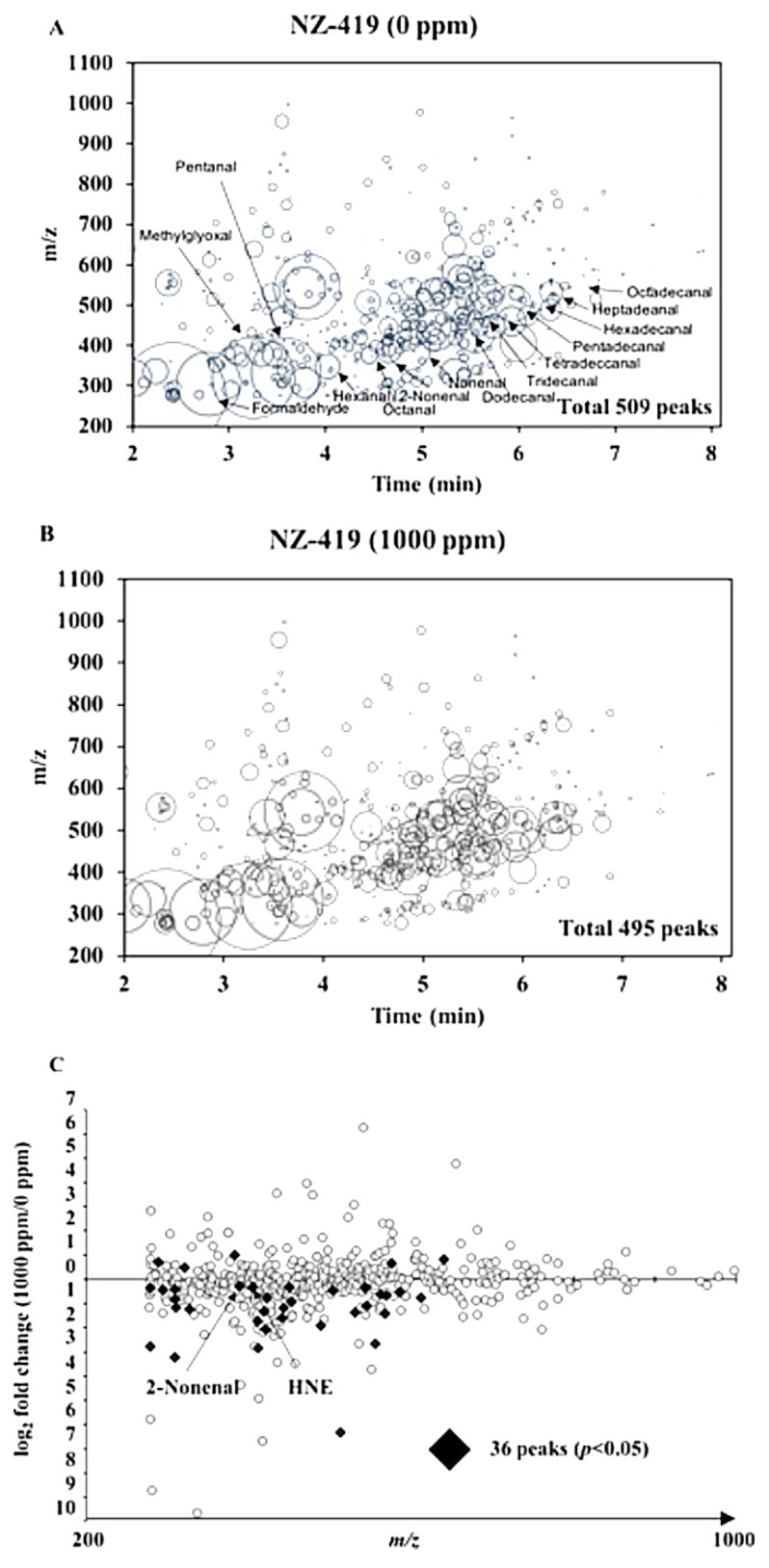
Reactive carbonyl species (RCs) maps plotting free RCs detected in the serum samples of Min mice. All free RCs detected in the serum samples taken from 0 (**A**) or 1000 (**B**) ppm NZ-419-treated Min mice are shown. The RCs are plotted as circles as a function of their retention times (horizontal axis) and *m*/*z* values (vertical axis). The areas of the circles represent the intensities of the peaks of the RCs relative to that of the internal standard (IS). The RCs abbreviations are listed in the Materials and Methods section. (**C**) The comparative RCs profiles of the serum samples from 1000 ppm NZ-419-treated Min mice. The closed diamonds indicate that the RCs levels were significantly different between non-treated and NZ-419-treated mice (*p* < 0.05).

To confirm the effects of NZ-419 on oxidative stress, we used liquid chromatography/mass spectrometry (LC/MS) and measured the levels of reactive carbonyl species (RCs) in the serum of Min mice treated with or without NZ-419. We believe that effects of NZ-419 have reached a plateau at the dose of 500 ppm. Thus, afterwards, we used the 1000 ppm treated sample to obtain enough difference. LC/MS detected 509 and 495 peaks in the samples taken from non-treated and 1000 ppm NZ-419-treated Min mice, respectively (Figure 5A,B). Of the 495 peaks detected, the levels of 305 peaks were lower in NZ-419-treated mice than in the non-treated mice, respectively, with the levels of 31 peaks being significantly lower, including 2-Nonenal and HNE (*p* < 0.05) (Figure 5C).

### 3.5. Suppression of mRNA Levels of Proliferation-Related Factor in Intestinal Polyp of Min Mice by NZ-419

**Figure 6:**
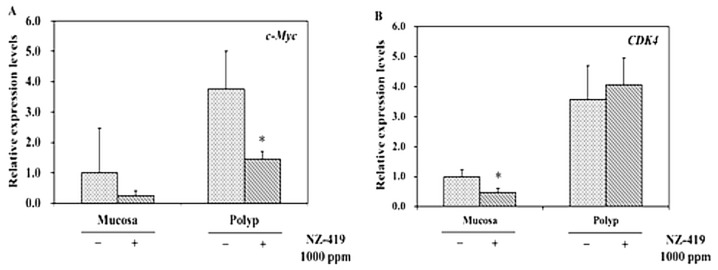
Suppression of *c-Myc* and *Cdk4* in non-polyp intestinal mucosa or/and polyp parts of Min mice treated or not with 1000 ppm NZ-419. The mice received diets containing NZ-419 at the indicated doses for 8 weeks. Quantitative real-time PCR analyses were performed to determine the *c-Myc* (**A**) and *Cdk4* (**B**) mRNA expression levels in the polyps and non-polyp intestinal mucosa of Min mice. The data are normalized to GAPDH and presented as the means ± SD (*n* = 3). The basal mRNA expression level in the control was set as 1.0. * *p* < 0.05 vs. 0 ppm.

To clarify the suppressive effects of NZ-419 on the target genes downstream of Tcf/LEF or cell cycle regulatory-related factors, the levels of these genes in the intestinal polyp and non-polyp parts of Min mice were evaluated. Treatment with 1000 ppm NZ-419 for 8 weeks significantly reduced *c-Myc* mRNA expression levels in polyp parts to 61% (*p* < 0.05) of the untreated value and reduced *c-Myc* mRNA expression in non-polyp intestinal mucosa to 74% of the untreated value (not statistically significant) (Figure 6A). *Cdk4* mRNA expression levels in non-polyp intestinal mucosa were reduced by 53% compared with the values in untreated mucosa (Figure 6B). Treatment with 500 ppm NZ-419 failed to statistically significantly reduce *c-Myc* and *Cdk4* mRNA levels (data not shown).

### 3.6. Tendency of NZ-419 to Decrease Cell Growth in Intestinal Polyps

**Figure 7:**
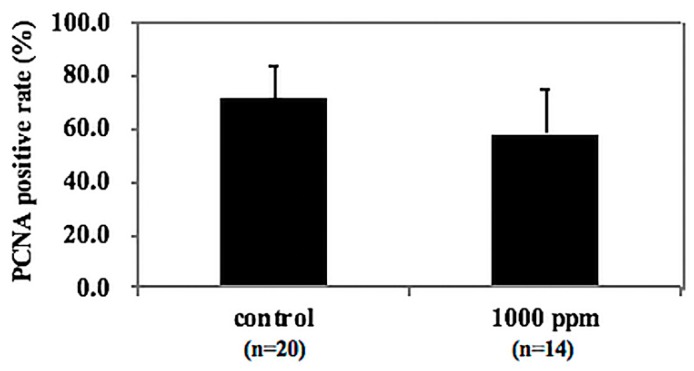
NZ-419 inhibited epithelial cell proliferation. Change in the ratio of proliferation cell nuclear antigen (PCNA)-positive cells in the intestinal polyps with NZ-419 treatment. Immunohistochemical staining for PCNA in the intestinal polyps of Min mice was performed in the basal diet group and 1000 ppm NZ-419-containing diet group. Calculations of the ratio of PCNA-positive cells are described in the Materials and Methods section. Bars indicate the SD (*n* = 7~11).

To investigate the effect of NZ-419 treatment on epithelial cell proliferation, intestinal polyps in Min mice were immunohistochemically stained with anti-proliferation cell nuclear antigen (anti-PCNA) antibody. As shown in Figure 7, an index of cell growth (number of PCNA stained nuclei/total nuclei in the polyp) revealed that administration of 1000 ppm NZ-419 showed a tendency to suppress cell proliferation in intestinal polyps in Min mice.

## 4. Discussion

In the present study, we demonstrated that NZ-419 eliminated ROS production in HCT116 cells after H_2_O_2_ stimulation and suppressed H_2_O_2_-induced Nrf2 promoter transcriptional activity in an in vitro setting. However, NZ-419 didn’t eliminate transcriptional activity without H_2_O_2_. This result shows NZ-419 might have ROS-independent effect. In an in vivo setting, NZ-419 suppressed intestinal polyp formation in Min mice. Suppression of the mRNA levels of the proliferation-associated factor *c-Myc* was observed in intestinal polyps of Min mice after NZ-419 treatment, but cell proliferation activity evaluated by immunohistochemical staining of PCNA showed only a weak suppression of cell proliferation in intestinal polyps in Min mice.

Consistent with a previous report that NZ-419 has ·OH scavenging ability, our results confirmed its potential. We used a specific oxidation-sensitive fluorescent probe, H_2_DCFDA, that detects hydrogen peroxide (H_2_O_2_) and cytosolic superoxide anions (·O^2−^). Hydrogen peroxide is a source of hydroxyl radical, and ·O^2−^ can be converted into hydroxyl radical through superoxide dismutase. In this experiment, we used NAC and 5-ASA as a positive control. The data from the positive control shows this assay is suitable for evaluating NZ-419 as a ROS scavenger. Besides effect for preventing CRC, they are famous for ROS scavenger. [20,21] NAC is a well-known anti-oxidant and also induce glutathione (GSH). On the other hand, 5-ASA extremely scavenges ROS, which supported in clinical evidence. [22] Our results indicated that NZ-419 reduced H_2_O_2_-induced oxidative stress. Compared to this result, cells only treated with NZ-419 didn’t decrease Nrf2 promoter transcriptional activity. Therefore, NZ-419 might only affected to the ROS-dependent effect. Nrf2 is transcription factor that functions as a crucial regulator of cellular antioxidant defense. Under oxidative stress conditions, Nrf2 is released from Kelch-like ECH-associated protein 1 (Keap1), which promotes the ubiquitination and degradation of Nrf2 [23]. The released Nrf2 translocates into the nucleus, forms heterodimers, and binds to antioxidant response elements to activate downstream genes, including phase II detoxifying enzymes [24].

To the best of our knowledge, this is the first report showing that radical scavenger can suppress intestinal polyp formation in mice. NZ-419 treatment tends to reduce total polyp development in the colon and small intestine, but this reduction is significant only in the middle portion of the small intestine. We failed to obtain significant effect on the reduction of polyp count in the 1000 ppm-treated group, but did in the 500 ppm-treated group. However, we consider the data biologically worthwhile because the number of the polyps observed in the 500 ppm and the 1000 ppm group was almost the same. We were unable to determine in this study why this was so. Other agents, such as indomethacin, a COX inhibitor; nimesulide, a COX-2 selective inhibitor; sesamol, a COX-2 suppressor; and apocynin, an NADPH oxidase inhibitor, have been shown to significantly reduce the number of intestinal polyps in the middle to distal parts of the intestine [25,26,27,28]. On the other hand, the lipoprotein lipase activator NO-1886, peroxisome proliferator-activated receptor ligands and erythromycin have been shown to mainly reduce the numbers of intestinal polyps in the proximal part of the small intestine [29,30,31]. The agents that suppressed intestinal polyp formation in the middle parts of the intestine in previous reports were antioxidative agents and anti-inflammatory agents, so the results of the antioxidative agent NZ-419 are consistent with previous reports. Thus, we surmised that its radical scavenger function plays a role in its effects on middle intestinal polyp development. We also evaluated the levels of oxidative stress markers, RCs, in the serum of NZ-419-treated Min mice, and find that the number of RCs were reduced by NZ-419 administration, that indicated NZ-419 worked in the mice.

We next investigated the tumor suppressive effects of NZ-419 by evaluating proliferation-associated factors in the mucosa and polyp parts of the small intestine of Min mice. We observed suppression of *c-Myc* mRNA levels in intestinal polyps of Min mice after NZ-419 treatment. *c-Myc* is an oncogene product and is related to cell cycle progression. Thus, it is reasonable to assume that NZ-419 suppresses *c-Myc* expression, resulting in a reduced number of PCNA-positive cells and resultant antiproliferation activity in neoplastic cells.

On the other hand, *c-Myc* is known as Nrf2 inhibitor that interferes with Nrf2 signaling and downstream target gene expression.

*c-Myc* interacts with the EpRE binding complex and increases the degradation of Nrf2. We do not know whether radical scavenging function of NZ-419 lowered *c-Myc* levels directly or lowered them indirectly through elevation of Nrf2 expression levels. Further experiments are needed to clarify the mechanisms.

NZ-419 possesses some cancer chemopreventive agent. It can administer orally, and it is under phase II trial. Moreover, NZ-419 seems to be an ideal cancer chemopreventive agent, as it meets several criteria: (I) having a convenient dosing schedule, (II) being easily administered, (III) being low cost, and (IV) having very mild side-effects [32].

## 5. Conclusions

In conclusion, this study demonstrated that NZ-419 suppressed the development of intestinal polyps in Min mice, suggesting the utility of radical scavenger/antioxidants as a cancer chemopreventive agent. Further in vitro and in vivo experiments and clinical trials investigating the suppression of colon carcinogenesis by NZ-419 are needed to provide solid evidence for NZ-419 as a cancer chemopreventive agent. In addition, if NZ-419 were clearly proven to be effective for the prevention of CRC and any other cancers, the impact would be extremely high in the context of drug repositioning.

## Figures and Tables

**Table 1:** The number of intestinal polyps/mouse in Min mice treated with or without NZ-419.

Dose	No. of Mice	Small Intestine			Colon	Total
(ppm)		Proximal	Middle	Distal		
0	9	5.8 ± 2.9	11.7 ± 3.4	29.3 ± 5.4	0.7 ± 1.0	47.4 ± 5.2
500	9	3.9 ± 1.6	7.3 ± 2.9 *	27.0 ± 10.2	1.0 ± 0.9	39.2 ± 10.3
1000	8	5.1 ± 2.4	8.3 ± 2.8	25.6 ± 7.2	0.4 ± 0.7	39.4 ± 9.6

Data are presented as the means ± standard deviation (SD). Significantly different from the untreated control group at * *p* < 0.05.

## Abbreviations

5-ASA	5-aminosalicylic acid
CRC	colorectal cancer
COX	cyclooxygenase
EPA	eicosapentaenoic acid
FAP	familial adenomatous polyposis
FBS	fetal bovine serum
GAPDH	glyceraldehyde-3-phosphate dehydrogenase
GPCRs	G protein-coupled receptors
NAC	*N*-Acetyl-l-cysteine
Keap1	Kelch-like ECH-associated protein 1
Nrf2	nuclear factor erythroid-2 related factor 2
PCR	polymerase chain reaction
PUFAs	polyunsaturated fatty acids
PCNA	proliferating cell nuclear antigen
RCs	reactive carbonyl species
